# Electrochemical Growth of Copper Crystals on SPCE for Electrocatalysis Nitrate Reduction

**DOI:** 10.3390/nano14211704

**Published:** 2024-10-24

**Authors:** Roberta Farina, Giuseppe D’Arrigo, Alessandra Alberti, Giuseppe E. Capuano, Domenico Corso, Giuseppe A. Screpis, Maria Anna Coniglio, Guglielmo G. Condorelli, Sebania Libertino

**Affiliations:** 1 Istituto per la Microelettronica e Microsistemi—Consiglio Nazionale delle Ricerche (CNR—IMM), Strada VIII Z.I., 5, 95121 Catania, Italy or roberta.farina@phd.unict.it (R.F.); giuseppe.darrigo@imm.cnr.it (G.D.); alessandra.alberti@imm.cnr.it (A.A.); giuseppeemanuele.capuano@imm.cnr.it (G.E.C.); ma.coniglio@unict.it (M.A.C.); 2 Dipartimento di Scienze Chimiche, Università degli Studi di Catania, viale A. Doria 6, 95125 Catania, Italy; guido.condorelli@unict.it; 3 Dipartimento di Scienze Mediche, Chirurgiche e Tecnologie Avanzate “G.F. Ingrassia”, Università degli Studi di Catania, via S. Sofia 87, 95123 Catania, Italy; giuseppe.screpis@studium.unict.it

**Keywords:** copper micro-flowers, electrochemical deposition, cyclic voltammetry, nitrate reduction

## Abstract

Copper is efficient, has a high conductivity (5.8 × 10^7^ S/m), and is cost-effective. The use of copper-based catalysts is promising for the electrocatalytic reduction of nitrates. This work aims to grow and characterize copper micro-crystals on Screen-Printed Electrodes (SPEs) for NO_3_^−^ reduction in water. Copper micro-crystals were grown by cyclic voltammetry. Different cycles (2, 5, 7, 10, 12, 15) of copper electrodeposition were investigated (potential ranges from −1.0 V to 0.0 V, scan rate of 0.1 V s^−1^). Electrodeposition generated different morphologies of copper crystals on the electrodes, as a function of the number of cycles, with various performances. The presence of numerous edges and defects in the copper micro-crystal structures creates highly reactive active sites, thus favoring nitrate reduction. The manufactured material can be successfully employed for environmental applications.

## 1. Introduction

NO_3_^−^ contamination in water sources is a serious environmental issue, as elevated nitrate concentration can pose health risks and harm ecosystems. For this reason, the development of efficient methods to detect and remove nitrates from water is essential. Electroreduction of NO_3_^−^ is a process of great scientific and industrial interest due to its importance in water purification, ammonia production, and the removal of environmental pollutants. The recent advancement of electrocatalysts that can selectively reduce waste nitrate to ammonia presents new opportunities for NO_3_^−^ processing, offering both environmental and economic benefits for sustainable NH_3_ synthesis [1,2,3,4,5]. Several studies have investigated the use of various catalytic materials to improve the efficiency and selectivity of this electrochemical reaction, such as Pt [6,7,8,9,10], Pd [8,9,10], Ag [11,12,13,14], Sn [15], Cu [15,16,17,18,19], Cu-Ni [20,21], Cu-Zn [22], and Cu-Sn [23]. Among the materials explored, copper has attracted particular attention due to its unique catalytic properties and relative abundance. Recent research has shown that copper can be used in various structural forms to improve the performance of electrochemical NO_3_^−^ reduction. For instance, Zhang et al. [24] reported copper nanowires to achieve the selective reduction of nitrate to ammonia with a high faradic efficiency. Similarly, Chen et al. [25] explored the use of copper nanoparticles supported on carbon electrodes, demonstrating a significant improvement in nitrate conversion compared to conventional catalysts. In the present work, we electrochemically deposited copper on top of commercially available screen-printed electrodes (SPEs). The used SPE cell is composed of three electrodes: the C working electrode (WE), Ag reference electrode (RE), and Pt counter electrode (CE). The carbon WE was chosen for its electrochemical stability and conductivity, which provides an ideal substrate for the deposition of copper micro-crystals, further improving the efficiency of the process. Furthermore, the use of SPEs as substrates simplifies the fabrication process and allows for easy scalability and reproducibility of the device [26,27]. Copper micro-flower crystals were obtained by performing different cyclic voltammetry (CV) cycles (2, 5, 7, 10, 12, 15). CV is a powerful electrochemical technique as it facilitates the formation of micro and nanostructured materials during the functionalization process. This can improve the electrode surface area, active sites for reaction, catalytic properties, and the efficiency of electron transfer processes [28,29]. The efficiency of NO_3_^−^ reduction was correlated to the specific crystallographic orientation of copper, with specific surfaces showing increased catalytic activity. Copper micro-flowers act as catalysts and offer numerous active sites for the nitrate electrochemical reduction.

The aim of the present work is to fully characterize the working electrode preparation method. Farina et al., in ref. [30], described the performance of a sensor based on five Cu electrodeposition cycles. In this work, we demonstrate why five cycles represent the optimal electrode fabrication method by careful chemical and morphological characterization followed by a performance evaluation of the different fabrication procedures. Morphological and structural characterizations were carried out on each prepared electrode using Scanning Electron Microscopy, X-ray diffraction, X-ray photoelectron spectroscopy, and Raman analysis, and the electrocatalytic performance in nitrate ions reduction of the different electrodes was studied.

## 2. Chemicals, Materials, and Electrode Fabrication

### 2.1. Chemicals and Instruments

All chemicals used in this study were of analytical grade and applied without further purification. Potassium chloride (KCl), copper sulfate pentahydrate (CuSO_4_·5H_2_O), and sodium nitrate (NaNO_3_) were sourced from Merck KGaA (Darmstadt, Germany). Milli-Q water (resistivity of at least 18.2 MΩ·cm), generated using the Simplicity UV system (Millipore, by Merck, Darmstadt, Germany), was utilized for all solution preparations. Screen-printed carbon electrodes (SPCE, code Ref. 150) were obtained from Metrohm DropSens s.r.l. (Origgio, VA, Italy). Copper electrodeposition and all electrochemical measurements were carried out on a Palmsens4 electrochemical workstation from PalmSens BV (C-PS4-BP.F2.10, GA Houten, The Netherlands). Scanning electron microscopy (SEM) analysis was conducted in SEM mode using a Raith 150 (Dortmund, Germany) e-beam lithography system. X-ray diffraction (XRD) patterns were collected using a Rigaku Smartlab system (Sevenoaks, UK), equipped with a rotating anode tube operating at 100 mA and a HyPix3000 detector, with Soller slits on both primary and secondary beams, using a step size of 0.02° at a scan speed of 4°/min. Raman spectra were obtained using the BRAVO handheld Raman spectrometer by Bruker (Billerica, MA, USA). The instrument employs a patented technique called Sequentially Shifted Excitation (SSE™), utilizing two lasers with wavelengths of 785 nm and 852 nm that work in tandem under the Duo Laser™ system to minimize fluorescence interference. XPS measurements were carried out using a Physical Electronics GMBH PHI 5800-01 spectrometer (Physical Electronics GmbH, Munich, Germany), equipped with a monochromatic Al-Kα source (1486.6 eV). A power beam of 300 W was employed to record the spectra. The assessment of surface species oxidation state and concentration was carried out with pass energies of 11.0 eV and 58.0 eV, respectively. The C1s peak at 284.8 eV was taken as a reference to set binding energies.

### 2.2. Working Electrode Preparation

Cu was deposited electrochemically on the carbon WE surface (4 mm diameter) using cyclic voltammetry. During the scan toward negative potentials, copper ions (Cu^2^⁺) in the solution are reduced to copper metal (Cu^0^) and deposited onto the working electrode surface. During the reverse scan, some of the deposited copper may oxidize again, depending on the potential range and scan rate. CV was performed in the potential range from −1.0 V to 0.0 V at a scan rate of 100 mV s^−1^ by scanning 2, 5, 7, 10, 12, and 15 times, using 0.1 M CuSO_4_∙5H_2_O in 0.1 M KCl as the supporting electrolyte [30]. This different process created several Cu morphological structures on the C surface.

## 3. Results and Discussion

### 3.1. WE Electrochemical Characterization

**Figure 1:**
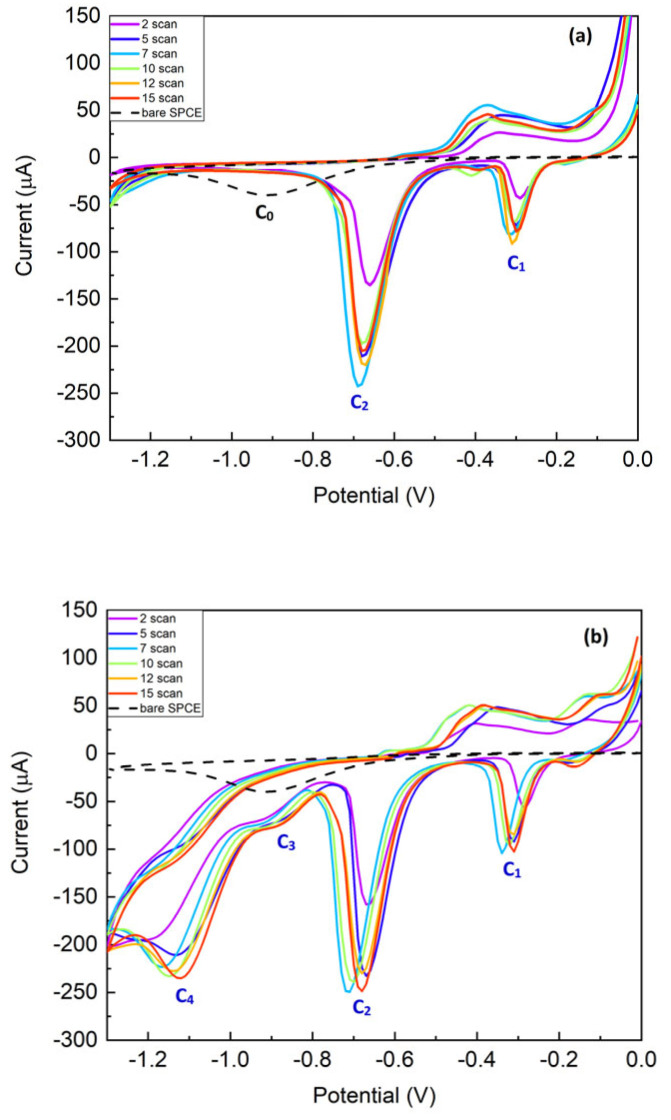
CV in 0.1 M KCl of (**a**) bare C electrode (black dash–dash trace) and modified Cu/C electrodes: 2 cycles (purple trace), 5 cycles (blue trace), 7 cycles (light blue trace), 10 cycles (green trace), 12 cycles (yellow trace), and 15 cycles (red trace); (**b**) bare C electrode (black dash–dash trace) and modified Cu/C electrodes: 2 cycles (purple trace), 5 cycles (blue trace), 7 cycles (light blue trace), 10 cycles (green trace), 12 cycles (yellow trace), and 15 cycles (red trace) in presence of nitrate concentration (1.6 mM NO_3_^−^).

The electrochemical characterization of the bare carbon electrode (SPCE) and Cu/C modified electrodes was performed in a 0.1 M KCl electrolyte solution, both in the absence (Figure 1a) and in the presence of nitrate concentration (1.6 mM NO_3_^−^) (Figure 1b). In the electrolyte solution, SPCE (black dash–dash trace) shows a C_0_ wave characteristic of the electrode, which smooths in the case of the copper-modified electrode. This wave remains unchanged in the presence of nitrate, demonstrating the inactivity of the bare carbon electrode towards nitrate ions. Figure 1a shows the voltammograms relative to the different scan depositions. They exhibit two cathodic peaks at −0.33 V (C_1_) and −0.75 V (C_2_). C_1_ and C_2_ peaks are associated with the reduction of Cu(I) and Cu(II), as illustrated by Equations (1) and (2). Their presence indicates the successful coating of the carbon WE.

(1) $$Cu\left(I\right)+e^{-}\to Cu,$$

(2) $$Cu\left(II\right)+2e^{-}\to Cu$$

In the presence of 1.6 mM (Figure 1b), two additional reduction peaks at −0.86 V (C_3_) and −1.08 V (C_4_) appear in the cyclic voltammogram at the Cu/C modified electrodes. Waves C_3_ and C_4_ denote the diffusion currents related to the reduction of NO_3_^−^ and NO_2_^−^ [31]. This reaction is illustrated by Equations (3) and (4).

(3) $${NO}_{3}^{-}+H_{2}O+{2e}^{-}\to {NO}_{2}^{-}+2{OH}^{-},$$

(4) $${NO}_{2}^{-}+{5H}_{2}O+{2e}^{-}\to {NH}_{3}+7{OH}^{-}.$$

The obtained results agree with those reported by Inam et al. [32] and Lotfi Zadeh Zhad et al. [33].

### 3.2. Structural Analysis

**Figure 2:**
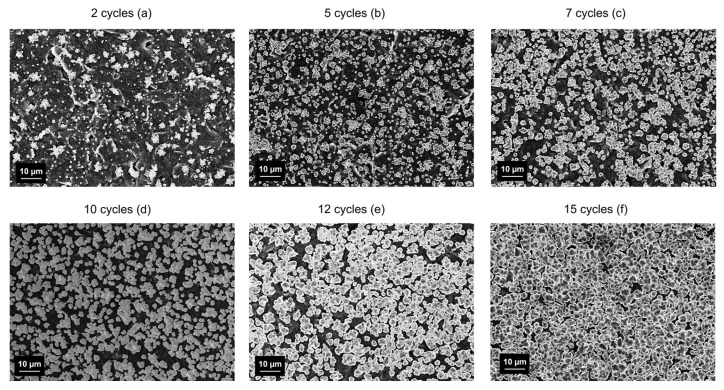
SEM images of carbon WE modified with (**a**) 2, (**b**) 5, (**c**) 7, (**d**) 10, (**e**) 12, and (**f**) 15 cycles of Cu electrodeposition.

Scanning electron micrographs were acquired to investigate the difference among the electrodes modified with 2, 5, 7, 10, and 15 CV electrodeposition cycles. Figure 2a clearly shows that after two CV cycles, the Cu deposition is non-uniform all over the WE and the underneath C is still clearly visible. Instead, five cycles (Figure 2b) showed a uniform distribution of the Cu all over the carbon electrode surface. The micrograph image of the seven Cu deposition cycles shows a still-visible carbon surface covered by larger copper crystallites. Instead, 10, 12, and 15 cycles of Cu deposition show a barely visible carbon surface covered by almost-uniform and merged copper structures.

**Figure 3:**
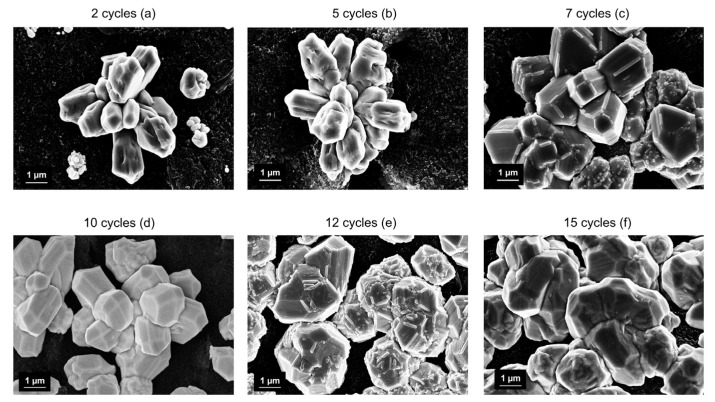
SEM magnification images of carbon WE modified with (**a**) 2, (**b**) 5, (**c**) 7, (**d**) 10, (**e**) 12, (**f**) 15 cycles of Cu electrodeposition.

Figure 3 shows a magnification of the copper structures achieved after different Cu electrodeposition cycles on the WE. Copper is deposited in well-defined flower-shaped crystalline structures which are already defined after two cycles (Figure 3a). After five cycles of Cu deposition, flower-shaped crystals with a specific orientation are formed through electrodeposition. (Figure 3b). In addition, the obtained Cu flowers provide an optimum surface-to-volume ratio of the electroactive surface, ensuring greater electrochemical accessibility for NO_3_^−^. The carbon surface remains visible, indicating that the electrode’s porosity has been maintained. Increasing the number of cycles, from seven electrodeposition cycles to as above, the samples show a good surface coverage. However, the copper crystal structures are swollen and have nucleation centers along the direction of the crystal’s orientation (Figure 3c). An increase in the number of electrodeposition cycles causes both the formation of large Cu clusters with a lower number of sites active for NO_3_^−^ reduction and the full coverage of the C surface (Figure 3d–f).

Therefore, for a larger number of deposition cycles, the crystallites already formed act as nucleation centers along the crystal orientation; this phenomenon promotes the formation of larger clusters that reduce the active surface area. The excess of copper ions causes significant stacking of Cu crystals, which greatly hinders mass transport and the electron transfer process.

The changes in crystal morphologies can lead to variations in electrocatalytic properties and specific surface areas. This provides different active sites and influences mass/charge transfer [2,34]. Electrocatalysts with specific morphologies can present distinct exposed crystal facets, enhancing both activity and selectivity for NO_3_⁻ reduction by altering the adsorption energies of reaction intermediates [35].

**Figure 4:**
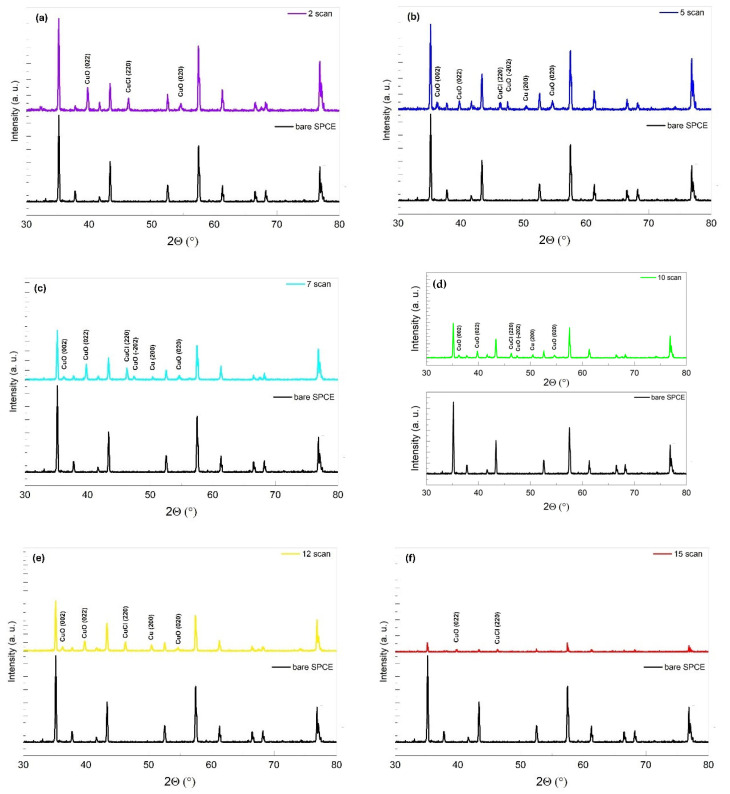
XRD Pattern for bare SPCE (black trace) and (**a**) modified electrode with 2 Cu electrodeposited cycles (purple trace); (**b**) modified electrode with 5 Cu electrodeposited cycles (blue trace); (**c**) modified electrode with 7 Cu electrodeposited cycles (light blue trace); (**d**) modified electrode with 10 Cu electrodeposited cycles (green trace); (**e**) modified electrode with 12 Cu electrodeposited cycles (yellow trace); (**f**) modified electrode with 15 Cu electrodeposited cycles (red trace).

The electrodes modified with the various deposition cycles were analyzed by X-ray diffraction to assess their crystallographic structure. Figure 4 presents a comparison of the XRD patterns for the unmodified SPCE electrode (black trace) and the modified electrodes. The Cu/C electrode modified with two cycles (purple trace in Figure 4a) shows peaks at 2θ positions of 39.76°, 47.46°, and 54.60°, corresponding to the Bragg reflections of crystalline CuO(002), CuCl(220), and CuO(020), respectively. The pattern for the Cu/C electrode modified with five cycles (blue trace in Figure 4b) shows a peak at the 2θ position of 46.3°, attributed to CuCl(220), and peaks at 2θ values of 36.18°, 39.76°, and 47.46°, corresponding to CuO(002), CuO(022), and CuO(-202), respectively. Additionally, peaks at the 2θ position of 50.48° and 74.13° are observed, corresponding to the Bragg reflections of crystalline Cu(200) and Cu(220). Patterns of electrodes of Cu/C after seven cycles (light blue trace) and ten cycles (green trace) show the pattern of the modified electrode of Cu/C after five cycles (Figure 4c,d).

The absence of a peak relative to the crystalline form of CuO(−202) is observed in the pattern of the Cu/C electrode modified after 12 cycles (yellow trace in Figure 4e). Finally, the modified electrode pattern after 15 cycles (red trace) shows only the peaks related to the CuO(022) and CuCl(220) crystal structures (Figure 4f). Different orientations have different surface energies, which affect the thermodynamic stability, the ability to adsorb nitrate molecules, and the reactivity. Surfaces with lower surface energies tend to be more stable but less reactive, while those with higher energies may be more reactive. Different crystallographic orientations provide varying densities of active sites, affecting the number of sites available for the catalytic reaction. A surface with a greater density of exposed copper atoms can more efficiently adsorb nitrates. Metallic copper is effective in promoting nitrate reduction due to its excellent electrical conductivity and abundance of conduction electrons. In contrast, the presence of chlorine can adversely affect the nature of the active sites and their interaction with nitrates [36].

The obtained XRD patterns confirm the successful deposition of crystalline Cu on the surface of the carbon working electrode. This observation is consistent with previous studies by Inam et al. [32] and Chen et al. [29], which demonstrated that copper electrocrystallization is influenced by the surface energies of different crystallographic planes. Consequently, XRD measurements provide insight into not only metallic copper but also other copper compounds present in the sample. Their formation is associated with anion-induced growth along specific copper crystal planes, optimizing the exposure of active sites and enhancing synergistic catalytic efficiency.

Raman analysis of the SPCE after 2, 5, 7, 10, and 15 CV copper electrodeposition cycles was performed. The spectra of the bare carbon electrode show no detectable peaks. In contrast, the modified electrodes exhibit two peaks at 518 and 622 cm⁻^1^, characteristic of copper oxides, corresponding to the symmetric vibrations of Cu-O bonds and vibrational modes associated with the CuO crystal lattice (Figure S1). It is worth remembering that Raman does not detect metallic Cu. The Raman spectra suggest an increase in copper oxide presence on the electrode surface as the number of functionalization cycles increases, likely due to progressive oxidation or greater CuO incorporation into the electrode surface.

X-ray photoelectron spectroscopy was employed to assess the samples’ surface chemical composition and the component species’ chemical environment [37,38,39]. As expected, the reference sample does not show the Cu peaks, which appear once the electrodeposition starts. The two main pieces of information that can be derived from the data are as follows: (i) the copper is present mainly in its metallic form and (ii) a component of CuO is present in the sample. The Cu2p characteristic peaks at 952.50 eV and 933 eV are slightly shifted, indicating the presence of other chemical bonds. Moreover, it is possible to observe that the typical CuO XPS peaks at 965 eV and 945 eV start to appear in all processed samples.

### 3.3. Performance of Electrochemical Electrodes for NO_3_^−^ Reduction

**Figure 5:**
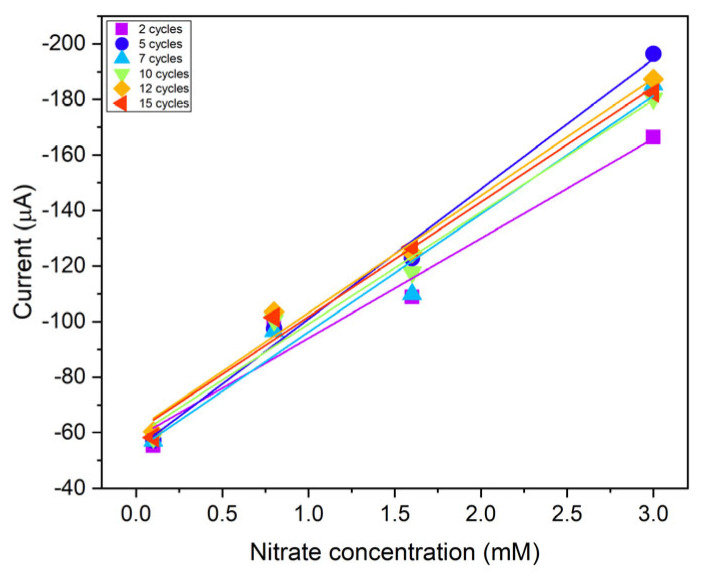
The calibration curves of the electrodes obtained after different electrodeposition cycles as a function of the nitrate concentration (0.1, 0.8, 1.6, and 3 mM): 2 cycles (violet squares), 5 cycles (blue dots), 7 cycles (light blue triangles), 10 cycles (green triangles), 12 cycles (yellow squares), and 15 cycles (red triangles). The points indicate the maximum cathodic current peak at −0.86 V, while the solid lines, plotted using the same data point color, are the linear best fit of the respective data.

To study the electrochemical analytical performances of the different electrodes, linear sweep voltammetry was used to detect NO_3_^−^ in a 0.1 M KCl electrolyte solution. Each electrode was examined in four solutions with increasing NO_3_^−^ concentrations (0.1, 0.8, 1.6, and 3 mM). Figure 5 shows the reduction current peak at −0.86 V for the six developed electrodes plotted against NO_3_^−^ concentration.

Electrodes show different sensitivities in nitrate electroreduction as a function of the deposition process. Table 1 shows the measured sensitivity for each electrode. The results show that five CV cycles provide the best result in terms of sensitivity and efficiency in nitrate reduction. In a previous study, it was found that the reaction occurring at the electrode is an irreversible, diffusion-controlled electron transfer process. The energy levels of the d-orbitals in modified copper SPCE are well-aligned with the molecular orbitals of NO_3_^⁻^, facilitating efficient electron transfer [30]. The micro-flower structure favors this process, since the catalytic activity in the electroreduction of nitrates is predominantly carried out on the tips of the well-defined structures of Cu crystals. They operate as active sites because there are more exposed copper atoms at the edges of the crystal than on a curved surface. These atoms, being less engaged in bonds with other Cu atoms, are more reactive and thus able to interact more easily with NO_3_^−^ molecules. As a result, the tips improve the local diffusion of nitrate ions, reducing the mass-limiting effect that can occur on rounder surfaces where the transport of reactants to the surface is less effective.

Using the Randles–Ševčík equation (Equation (5)), the relative surface areas of the six different electrodes were calculated (Table 1).

(5) $$i_{p}=-2.69\times 10^{5}n^{\frac{3}{2}}{AD}_{0}^{\frac{1}{2}}\nu^{\frac{1}{2}}C.$$

Here, *i_p_* is the peak current of the redox reaction, *n* is the number of electrons of the reaction (2 for NO_3_^−^), *A* is the active surface area of the WE (cm^2^), *D_o_* is the diffusion coefficient of the redox species (2.0 × 10^−6^ cm^2^ s^−1^ for NO_3_^−^), v is the scan rate used to collect the voltammogram (V s^−1^), and *C* is the NO_3_^−^ concentration (mol cm^−3^) [40,41,42].

**Figure 6:**
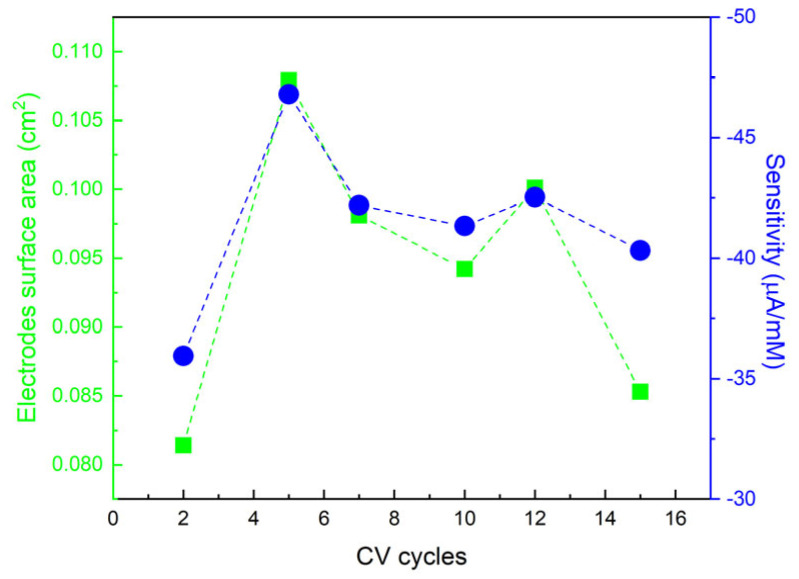
The sensitivity (blue dots related to the vertical right axis) and calculated electrode surface areas (green squares related to the vertical left axis) for 3 mM NO_3_^−^, with both as a function of the electrodes Cu functionalization cycles.

A 100% increase in surface area was seen for different copper-modified electrodes compared to the bare SPCE. Figure 6 shows that the calculated sensitivity for each electrode is in agreement with the corresponding calculated surface area.

The results confirmed that the electrode modified by five cycles of deposition is the finest in NO_3_^−^ reduction among the six electrodes manufactured. Next are the electrodes modified with 12 cycles, 7 cycles, 10 cycles, 15 cycles, and finally the electrode modified with 2 cycles. The obtained sensitivities and surface area values can be explained by the morphological structure of the deposited copper and the carbon coating of the electrode. A higher surface area value is indicative of a highly active surface formed by well-defined structures, suggesting a controlled growth of copper with higher reactivity. The formation of nucleation centers on already formed crystals suggests more complex growth, with new copper layers forming on top of the existing ones (modified electrodes with 7 and 12 CV cycles). This is consistent with a structure that increases the active area, but not as much as the more defined structures formed with short cycles (two and five CV cycles). The formation of large, ill-defined clusters leads to a reduction in the active area, as the clusters tend to aggregate and reduce the area accessible for nitrate reduction. Thus, the decrease in the active area is consistent with the formation of coarser and less electrochemically efficient structures (modified electrodes with 10 and 15 CV cycles). This produces a decrease in sensitivity and electrode performance. Finally, the electrode modified with two CV cycles shows the worst sensitivity and a lower surface area because, although the deposited copper structures have a morphology with a defined grain structure, the electrode coating is poor.

The best device (five cycles of copper electroreduction) was also tested in real conditions by using tap water. In particular, in Ref. [30], we constructed a calibration curve in tap water and compared it with NO_3_^−^ calibration using milliQ water. The data also demonstrate that no interference is measurable in real water samples.

A cycling test and reusability test were performed on the modified electrode (five cycles) that showed better performance in nitrate electroreduction. The electrode exhibited stable behavior through the tenth measurement, showing a 5% reduction in the amplitude of the NO_3_^−^ cathodic peak relative to the first measurement (Figure S2).

To perform the reusability test, the electrodes were stored in a nitrogen atmosphere and reused after storage for up to 80 h. The results show that during the first few hours, there is a rapid decrease in current, and, consequently, a decline in electrode performance, followed by a stabilization phase. This behavior can be explained by the fact that, once stored under nitrogen, the electrode undergoes changes in its surface properties, which initially lead to a sharp drop in its electrochemical response (Figure S3).

The reduction in the amplitude of the NO_3_^−^ cathodic peak after usage is due to a partial modification of the micro-flower structure.

## 4. Conclusions

In this work, catalytic electrodes were developed for nitrate reduction. Using several cyclic voltammetry cycles (2, 5, 7, 10, 12, 15), copper was electrodeposited on the electrodes, giving rise to different crystal structures. The copper structures formed on each electrode were analyzed. The functionalization process resulted in a 100% increase in surface area for the six copper-modified electrodes compared to the bare carbon electrode. Using linear voltammetry, the performance of the various electrodes was studied. On the electrode modified with five CV cycles, a flower-like copper structure was obtained. The presence of numerous edges and defects in the copper micro-flower structures creates highly reactive active sites, favoring nitrate reduction. Therefore, the electrode modified with five cycles of CV deposition showed the best performance in catalyzing the NO_3_^−^ reduction reaction in terms of sensitivity. This study provides a way to construct an electrochemical electrode based on catalytic copper micro-flowers that is easy to fabricate, inexpensive, and efficient for nitrate reduction in water.

## 5. Patents

Farina, R.; Libertino S. Nitrates electrocatalytic detection in water by copper micro-flowers, Italian patent requested (Italian MIMIT) on 11 March 2024, file n. 102024000005344.

## Figures and Tables

**Table 1:** Sensitivity of different electrodes and calculated electrodes’ surface area.

Electrodes	Sensitivity	Surface Area (cm^2^)
Bare C	-	0.0197
2 cycles	35.94 μA/mM	0.0854
5 cycles	46.80 μA/mM	0.1079
7 cycles	42.19 μA/mM	0.0981
10 cycles	41.33 μA/mM	0.0942
12 cycles	42.53 μA/mM	0.1001
15 cycles	40.32 μA/mM	0.0853

## Data Availability

The raw data supporting the conclusions of this article are included in the paper figures and tables.

## Supplementary Materials

The following supporting information can be downloaded at: https://www.mdpi.com/article/10.3390/nano14211704/s1, Figure S1: Raman spectra of SPCEs modified with (a) 2, (b) 5, (c) 7, (d) 10, (e) 12, (f) 15 cycles of Cu electrodeposition. Figure S2. Cycling test for 5 cycles Cu/C modified electrode. The reduction peak current of the NO_3_^−^ was acquired from the same sample by repeating the measurement 10 times. The percentage reported in the Figure is the difference between the successive measurements from the first one. Figure S3. Reusability test for 5 cycles Cu/C modified electrode after a time of storage in N_2_ atmosphere.
