# Supplementary material for: Electrochemical Growth of Copper Crystals on SPCE for Electrocatalysis Nitrate Reduction
Source: Nanomaterials (Basel). 2024 Oct 24;14(21):1704. doi: 10.3390/nano14211704 (PMC11547369; doi:10.3390/nano14211704)
Supplement: Supplementary file 1 [file nanomaterials-14-01704-s001.zip › nanomaterials-3201103-supplementary.pdf]

Supplementary Materials

Electrochemical growth of copper crystals on SPCE for electrocatalysis nitrate reduction

Roberta Farina[1,2], Giuseppe D'Arrigo[1], Alessandra Alberti[1], Giuseppe E. Capuano[1], Domenico Corso*[1], Giuseppe A. Screpis[3], Maria Anna Coniglio[3,1], Guglielmo G. Condorelli[2], Sebania Libertino*[1]

[1]Consiglio Nazionale delle Ricerche – Istituto per la Microelettronica e Microsistemi (CNR – IMM), strada VIII Z.I., 5, 95121 Catania, Italia;

[2]Università degli studi di Catania, Dipartimento di Scienze Chimiche, viale A. Doria 6, 95125 Catania, Italia.

[3]Università degli studi di Catania, Dipartimento di Scienze Mediche, Chirurgiche e Tecnologie Avanzate "G.F. Ingrassia", via S. Sofia 87, 95123 Catania, Italia

*Correspondence: sebania.libertino@cnr.it; domenico.corso@imm.cnr.it

Morphological Characterization

Raman spectra were acquired in the range from 300 to 2000 cm^{-1}, with the following parameters setting manual integration time: 1200 ms and 2 scans for each measurement.

Raman analysis of the modified electrodes with 2, 5, 7, 10, and 15 CV copper electrodeposition cycles was performed. The spectra of the bare carbon electrode show no peaks. In contrast, the spectra of the modified electrodes exhibit two peaks at 518 and 622 cm^{-1}, which are characteristic of copper oxides and relate to the symmetrical vibrations of Cu-O bonds as well as the vibrational modes associated with the CuO crystal lattice (Figure S1). Raman spectra indicate an increase in copper oxides on the electrode surface as functionalization cycles increase, probably due to a progressive oxidation process or increased CuO incorporation into the electrode surface.

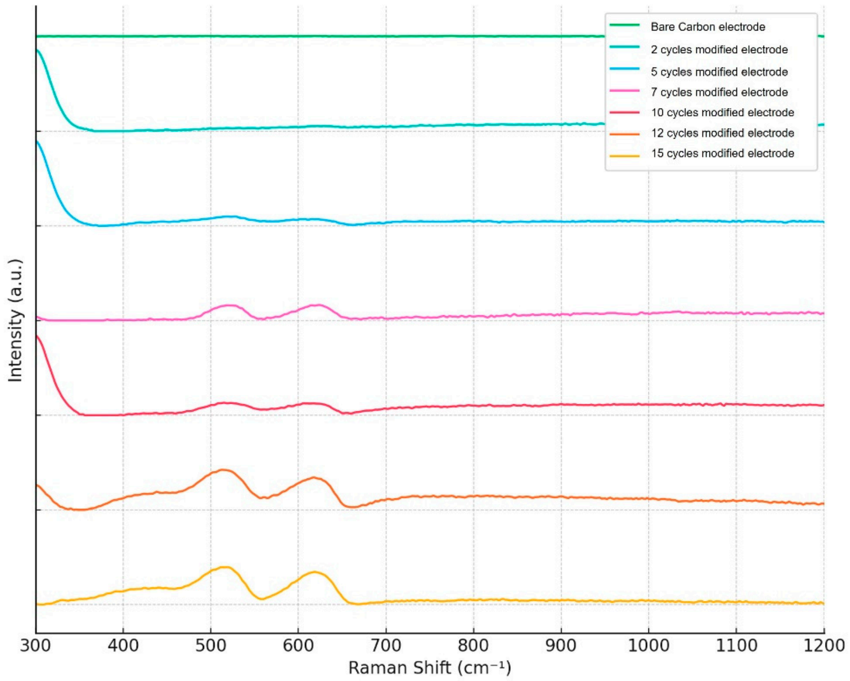

Figure S1. Raman spectra of SPCEs modified with (a) 2, (b) 5, (c) 7, (d) 10, (e) 12, (f) 15 cycles of Cu electrodeposition.

Electrochemical sensors performance for nitrate ions reduction

Cycling test and reusability test were performed. The data have been acquired on the solution containing 2mM NO_3^- for the electrode that showed the best performance (5 ycles).

The electrode had a stable behavior up to the tenth measurement. The repeatability test was carried out using the same electrode (10 consecutive measurements) in the same NO_3^- (2 mM) solution (Figure S2). The calculated relative standard deviation were: 0%, 0.8%, 1.37%, 2.61%, 3.18%, 3.74%, 4.15%, 4.63%, 5.10%, and 5.63%, respectively, for the four NO_3^- concentrations, demonstrating good repeatability.

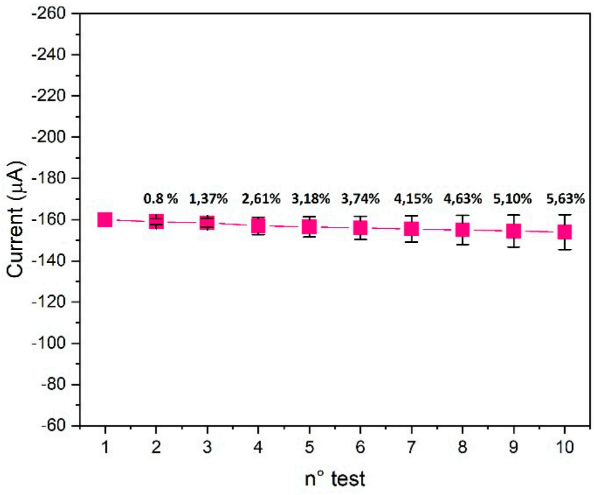

Figure S2. Cycling test for 5 cycles Cu/C modified electrode. The reduction peak current of the NO_3^- was acquired from the same sample by repeating the measurement 10 times. The percentage reported in the Figure is the difference between the successive measurements from the first one.

To perform reusability test, the electrodes were stored in a nitrogen atmosphere and reused after storage for up to 80 hours (Figure S3). We plot the current change as a function of the measurement time expressed in hours. The results show that during the first few hours, there is a rapid decrease in current, and consequently, a decline in electrode performance, followed by a stabilization phase. This behavior can be explained by considering that, once stored under nitrogen, the electrode changes its surface properties, which initially leads to a sharp drop in its electrochemical response.

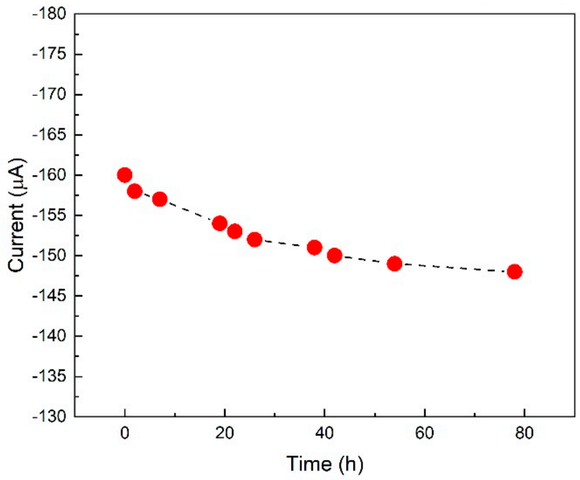

Figure S3. Reusability test for 5 cycles Cu/C modified electrode after a time of storage in N_2 atmosphere.
